# Field study site selection, species abundance and monthly distribution of anopheline mosquitoes in the northern Kruger National Park, South Africa

**DOI:** 10.1186/1475-2875-13-27

**Published:** 2014-01-24

**Authors:** Givemore Munhenga, Basil D Brooke, Belinda Spillings, Leyya Essop, Richard H Hunt, Stephen Midzi, Danny Govender, Leo Braack, Lizette L Koekemoer

**Affiliations:** 1 Centre for Opportunistic, Tropical and Hospital Infections, National Institute for Communicable Diseases, Private Bag X4, Sandringham, Johannesburg 2131, South Africa; 2 Wits Research Institute for Malaria, School of Pathology, Faculty of Health Sciences, University of the Witwatersrand, Johannesburg, South Africa; 3 Shangoni Section, Kruger National Park, Private Bag X402, Skukuza 1350, South Africa; 4 Scientific Services, South African National Parks, Private Bag X402, Skukuza 1350, South Africa; 5 Department of Paraclinical Sciences, Faculty of Veterinary Science, University of Pretoria, Private Bag X04, Onderstepoort 0110, South Africa; 6 Zoonoses Research Unit, Faculty of Health Sciences, University of Pretoria, Pretoria 0007, South Africa

**Keywords:** *Anopheles arabiensis*, Malaria vector control, Sterile insect technique, Kruger National Park, South Africa

## Abstract

**Background:**

Knowledge of the ecology and behaviour of a target species is a prerequisite for the successful development of any vector control strategy. Before the implementation of any strategy it is essential to have comprehensive information on the bionomics of species in the targeted area. The aims of this study were to conduct regular entomological surveillance and to determine the relative abundance of anopheline species in the northern Kruger National Park. In addition to this, the impact of weather conditions on an *Anopheles arabiensis* population were evaluated and a range of mosquito collection methods were assessed.

**Methods:**

A survey of *Anopheles* species was made between July 2010 and December 2012. Mosquitoes were collected from five sites in the northern Kruger National Park, using carbon dioxide-baited traps, human landing and larval collections. Specimens were identified morphologically and polymerase chain reaction assays were subsequently used where appropriate.

**Results:**

A total of 3,311 specimens belonging to nine different taxa was collected. Species collected were: *Anopheles arabiensis* (n = 1,352), *Anopheles quadriannulatus* (n = 870), *Anopheles coustani* (n = 395), *Anopheles merus* (n = 349), *Anopheles pretoriensis* (n = 35), *Anopheles maculipalpis* (n = 28), *Anopheles rivulorum* (n = 19), *Anopheles squamosus (*n = 3) and *Anopheles rufipes* (n = 2). Members of the *Anopheles gambiae* species complex were the most abundant and widely distributed, occurring across all collection sites. The highest number of mosquitoes was collected using CO_2_ baited net traps (58.2%) followed by human landing catches (24.8%). Larval collections (17%) provided an additional method to increase sample size. Mosquito sampling productivity was influenced by prevailing weather conditions and overall population densities fluctuated with seasons.

**Conclusion:**

Several anopheline species occur in the northern Kruger National Park and their densities fluctuate between seasons. Species abundance and relative proportions within the *An. gambiae* complex varied between collection methods. There is a perennial presence of an isolated population of *An. arabiensis* at the Malahlapanga site which declined in density during the dry winter months, making this site suitable for a small pilot study site for Sterile Insect Technique as a malaria vector control strategy.

## Background

Malaria remains a major public health concern in South Africa [1]. Despite years of well-managed malaria vector control programmes, sporadic outbreaks continue to occur in the malarious areas in the northeast of the country. Various strategies have been used to combat malaria but vector control through indoor residual spraying (IRS) remains the most effective tool [1]. The application of IRS is becoming problematic due to the development of insecticide resistance in target malaria vector populations thereby compromising malaria control efforts [2,3]. In addition, IRS is less effective at controlling *Anopheles arabiensis,* which is responsible for low-level, seasonal malaria transmission in South Africa [1]. Additional strategies are therefore needed to target those vector populations that feed and rest outdoors and are unaffected by conventional IRS. These factors, as well as the South African government’s mandate to eliminate malaria transmission by 2018 [4], led to initiatives to investigate additional vector control interventions to supplement the existing strategies [5]. The use of the sterile insect technique (SIT) for vector control in South Africa is being assessed as one such additional intervention.

Before the implementation of any new or additional vector control intervention (as well as for SIT), it is essential to have comprehensive information on the bionomics of mosquitoes in the targeted area [6]. It is also necessary to assess the technical, operational and economic likelihood of the technique to avoid unnecessary wastage of resources [6,7]. A site targeted for mosquito SIT should have certain characteristics. Primarily, it should contain a single stable malaria vector population that is genetically homogenous, occurs at a relatively low density and is isolated from other vector populations of the same species [6,8,9]. In addition, other criteria such as easy accessibility to the site are advantageous [8].

There are very few sites in South Africa where stable anopheline populations can be found in large enough numbers to effectively study the potential of SIT as a local malaria vector control tool. It has previously been established that the Kruger National Park (KNP) supports an isolated, relatively large *An. arabiensis* population at Malahlapanga [10-12], a remote locality at which there are no control interventions. There is however limited information on the anopheline fauna of the Kruger National Park. It was therefore necessary to obtain baseline information on the species diversity, ecology and population dynamics of anopheline populations in the KNP as well as to evaluate entomological surveillance tools that could potentially be used to monitor these populations.

The aim of this project therefore was to assess species composition, seasonal occurrence and distributions of *Anopheles* mosquitoes in the northern Kruger National Park, as well as to investigate the suitability of various sites and collection methods so as to provide baseline data prior the onset of a larger pilot SIT project.

## Methods

### Mosquito sampling and sterile insect technique field site investigation

Two initial mosquito collections were undertaken in June and November 2010 with the aim of assessing the presence, abundance and seasonal distribution of *An. arabiensis* at Malahlapanga in the Kruger National Park (22°53’22.61”S; 31°02’22.48”E), a site historically known to support an *An. arabiensis* population [10-12]. Subsequent collections were performed monthly from four additional locations in the northern region of the Kruger National Park (September 2011 to December 2012) in order to determine the presence of *An. arabiensis* in four other sites. The sites investigated were Louis se gat (23°06’39.88”S; 31°27’24.90”E), Sirheni bush camp (22.94’93.80”S; 31.23’09.30”E), Mafayeni (23°00’47.08”S; 31°14’15.26”E) and Matiovila geothermal springs (23°00’29.14”S; 31°14’03.35”E).

**Figure 1:**
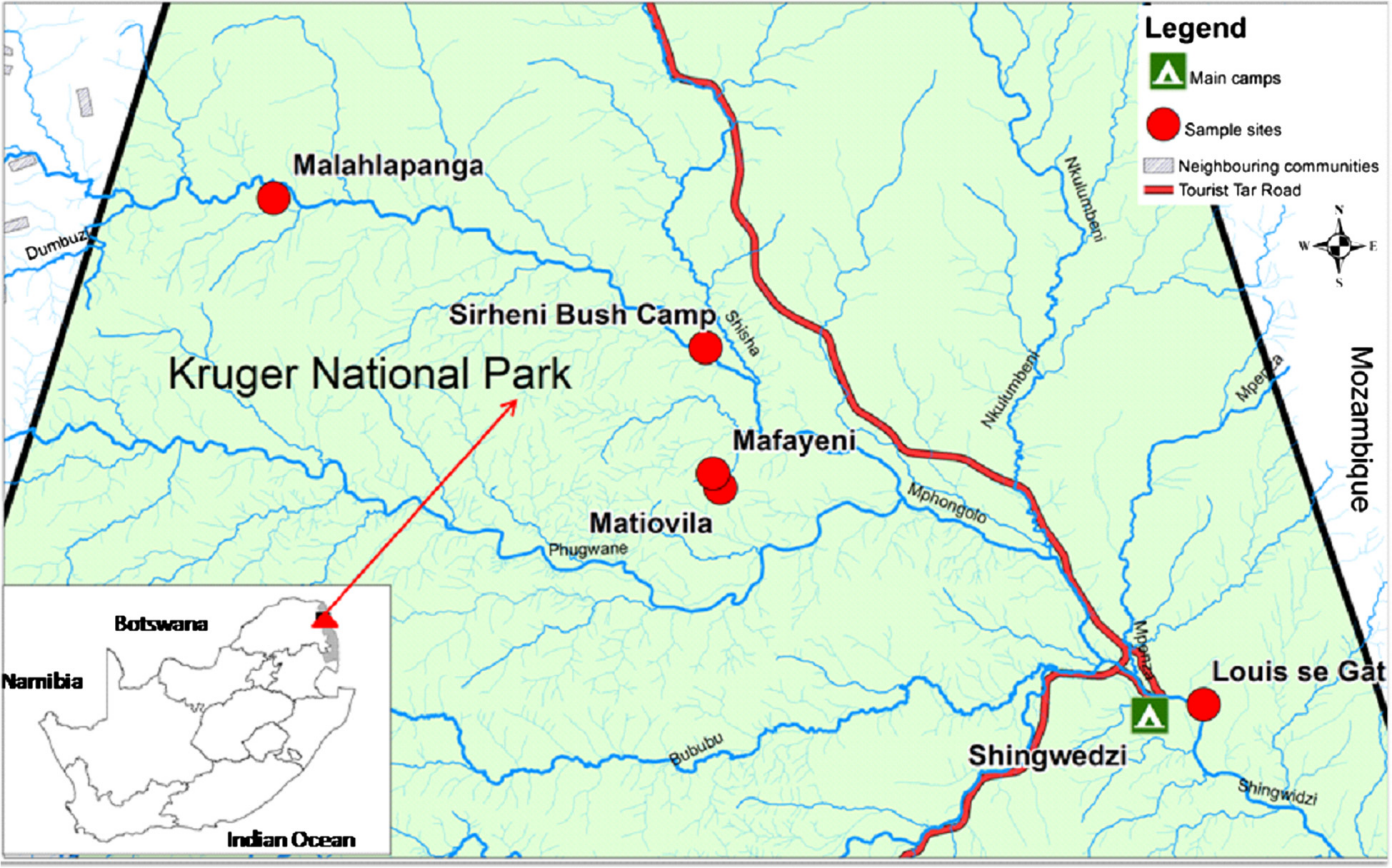
Map of northern Kruger National Park, South Africa, showing locations of sampling sites.

Malahlapanga is a freshwater geothermal spring situated in the northwestern region of the Kruger National Park (Figure 1). The spring is surrounded by *Colophospermum mopane* and *Acacia nigrescens* trees [10]. Warm water (~37°C) from the eye of the spring flows downstream, creating a wide wetland with a profusion of suitable breeding sites for mosquitoes. The spring supports a perennial, geographically isolated population of *An. arabiensis*[10,12]. Proliferation of mosquitoes is supported by abundant wildlife that uses the pan as a water source [12]. Louis se gat, Mafayeni, Matiovila, and Sirheni are comparatively similar to Malahlapanga and consist of Bush-Tree Savanna characterized by *C. mopane and Terminalia prunoides.* In addition, the Sirheni dam vicinity is rocky and has a geological formation of Archaean granite and Swaziland system which is characterized by Granite, Gneiss, Magmitite, Schist, Amphibolites and undifferentiated metamorphic rock with isolated tracts of *Terminalia sericea*.

Host-seeking female mosquitoes were collected by outdoor human landing catches and CO_2_ baited net traps between 18.00 and midnight as detailed in Munhenga *et al.*[13]. Due to the limitations of working in a National Park the aim of the larval collections was to increase the sample size of the collections. Larval collections were conducted at Sirheni, Malahlapanga, Matiovila and Mafayeni using a larval dipper (350 ml, 11.5 cm in diameter) (Mosquito Control Services and Suppliers, Roselle, ILL, USA) from at least ten different stagnant water bodies at each locality. Adult collections were done at Malahlapanga, Sirheni dam and Louis se gat but not at Matiovila and Mafayeni because these two sites are too remote and difficult to access at night. Mosquitoes were collected for a minimum of two consecutive nights for each sampling period. Collected adult mosquitoes were kept in gauze-covered paper cups and maintained in a humid box until their transportation to the laboratory in Johannesburg. Cotton wool pads soaked in a 10% sucrose solution were provided for each cup. Collected larvae were kept in containers and fed finely ground dog biscuits and brewer’s yeast (3:1). Field collected larvae were reared to adulthood for morphological identification. All samples were transported to the laboratory for further processing.

### Species identification

Collected anophelines were identified morphologically using appropriate keys [14,15]. Specimens positively identified as belonging to the *An. gambiae* complex and the *An. funestus* group were identified to species by polymerase chain reaction (PCR) [13,16]. *Anopheles coustani* group specimens were not identified to species level.

### Seasonal variation and the effect of environmental factors on mosquito catch productivity

Seasonal variations in mosquito catches were assessed through correlating total catches against seasons (ie, spring, summer, autumn, and winter). During each collection period environmental variables (temperature, rainfall, humidity, and wind speed) were recorded. Humidity and rainfall data were obtained from a weather station located in a tourist camp close to the sampling sites. Temperatures and wind speeds were obtained in real time from a Norwegian satellite based weather website [17].

### Salinity tests

A titration-based method adapted from Sinton & Kehar [18] was used to determine the salinity of water from Mafayeni and Matiovila. Briefly, 4 ml water samples from each of the sites were added to a flat-bottomed conical flask together with three drops of 5% potassium chromate. A solution of silver nitrate (9.58 g/l) was slowly added to this solution from a burette while continuously swirling the conical flask until an end point indicated by a persistent deep red flocculate of silver chromate was reached. The volume of silver nitrate needed to reach this end point was used to calculate the concentration of chloride present in the water sample. The chloride content was subsequently converted into weight of sodium chloride equivalent. These tests were conducted in order to help explain the presence of the salt-water breeder *An. merus* at these two study sites.

### Data analysis

Total mosquito catches, species collected, season of collection, collection method and environmental variables (temperature, humidity, rainfall, and wind speed) were recorded.

The relative frequency of each species was calculated against the total catch during the sampling period. Relative abundance of members of the *An. gambiae* complex was determined for each collection site. Trans-sectional species distribution was analysed using contingency tables.

Univariate and multivariate analyses were performed in statistix 8. Seasonal variation in mosquito catches was analysed using repeated ANOVA. Multiple regression analysis was used to explain the variation in total mosquito catches with respect to the following environmental variables (temperature, humidity and wind speed). Pearson correlation analysis was then used to assess the relationship between total catches and the environmental parameters. All statistical analyses were performed at 5% significance and comparisons between sites were only done during those months when collections were done at all sites.

## Results

### Species composition and abundance

Only data on the anophelines collected were analysed although large numbers of culicines and *Aedes* specimens were also collected. A total of 3,311 mosquitoes belonging to nine *Anopheles* species was collected from the five localities over a two-year sampling period. Of these, 3,053 were identified to species while 7.3% could not be identified. This might be due to incorrect morphological identification or human error. The CO_2_-baited tent traps accounted for 58.2% of mosquitoes collected, larval collections accounted for 24.8%, and human landing catches accounted for 17%. The species identified were *Anopheles gambiae* complex (*An. arabiensis*, *Anopheles quadriannulatus, Anopheles merus)*, *Anopheles funestus* group (*Anopheles rivulorum)*, *Anopheles coustani* group, *Anopheles maculipalpis*, *Anopheles squamosus, Anopheles pretoriensis* and *Anopheles rufipes. Anopheles coustani* group were not identified to species level.

**Figure 2:**
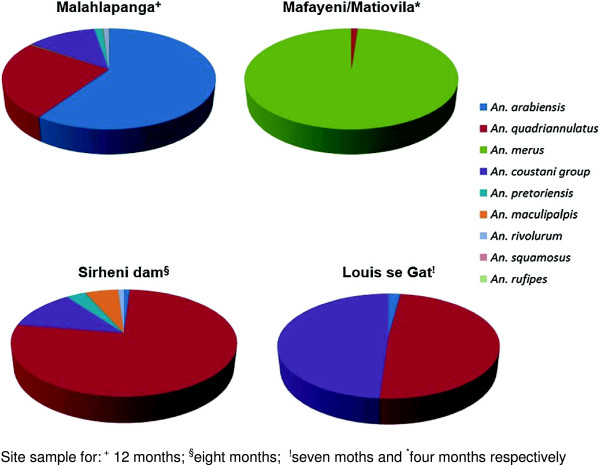
Relative abundance and geographical distribution of anophelines collected from five localities in northern Kruger National Park, South Africa, between July 2010 and December 2012.

Mosquito prevalence by species and their relative abundances are summarized in Table 1. The most widely distributed species was *An. quadriannulatus*. The other three common species in the pooled data were *An. arabiensis*, *An. maculipalpis* and *An. coustani* group. Specimens of each of these four taxa were found at three or more sampling sites (Figure 2). *Anopheles squamosus* and *An. rufipes* were the least prevalent with their occurrence limited to Malahlapanga.

**Table 1:** Summary of anopheline mosquitoes collected from northern Kruger National Park, South Africa, between July 2010 and December 2012

	**Total collected (N)**	**Relative frequency (%)**	Number of sites collected from*
*An. arabiensis*	1, 352	44.3	3
*An. quadriannulatus*	870	28.5	4
*An. merus*	349	11.4	2
*An. coustani* group	395	12.9	3
*An. pretoriensis*	35	1.1	2
*An. maculipalpis*	28	0.9	3
*An. rivulorum*	19	0.6	2
*An. squamosus*	3	0.1	1
*An. rufipes*	2	0.1	1

(N = number collected; % = percentage of total collection).

*For specific information on collection sites, see Figure 2.

Overall, *An. arabiensis* (44.3%) was the most abundant species, followed by *An. quadriannulatus, An. coustani* and *An. merus* which contributed 28.5%, 12.9% and 11.4% of the total catches respectively (Table 1). *Anopheles squamosus* and *An. rufipes* were the least abundant species contributing 0.1% each to the total collection.

### Species distribution

The species distribution patterns of anopheline mosquitoes collected are summarized in Figure 2. The largest numbers (75.4%) were collected from Malahlapanga and the lowest numbers (4.6%) were collected at Louis se gat. The greatest species diversity (nine out of nine species) was recorded at Malahlapanga followed by Sirheni (six out of nine species). Mafayeni had the lowest anopheline species diversity with only one species recorded.

**Figure 3:**
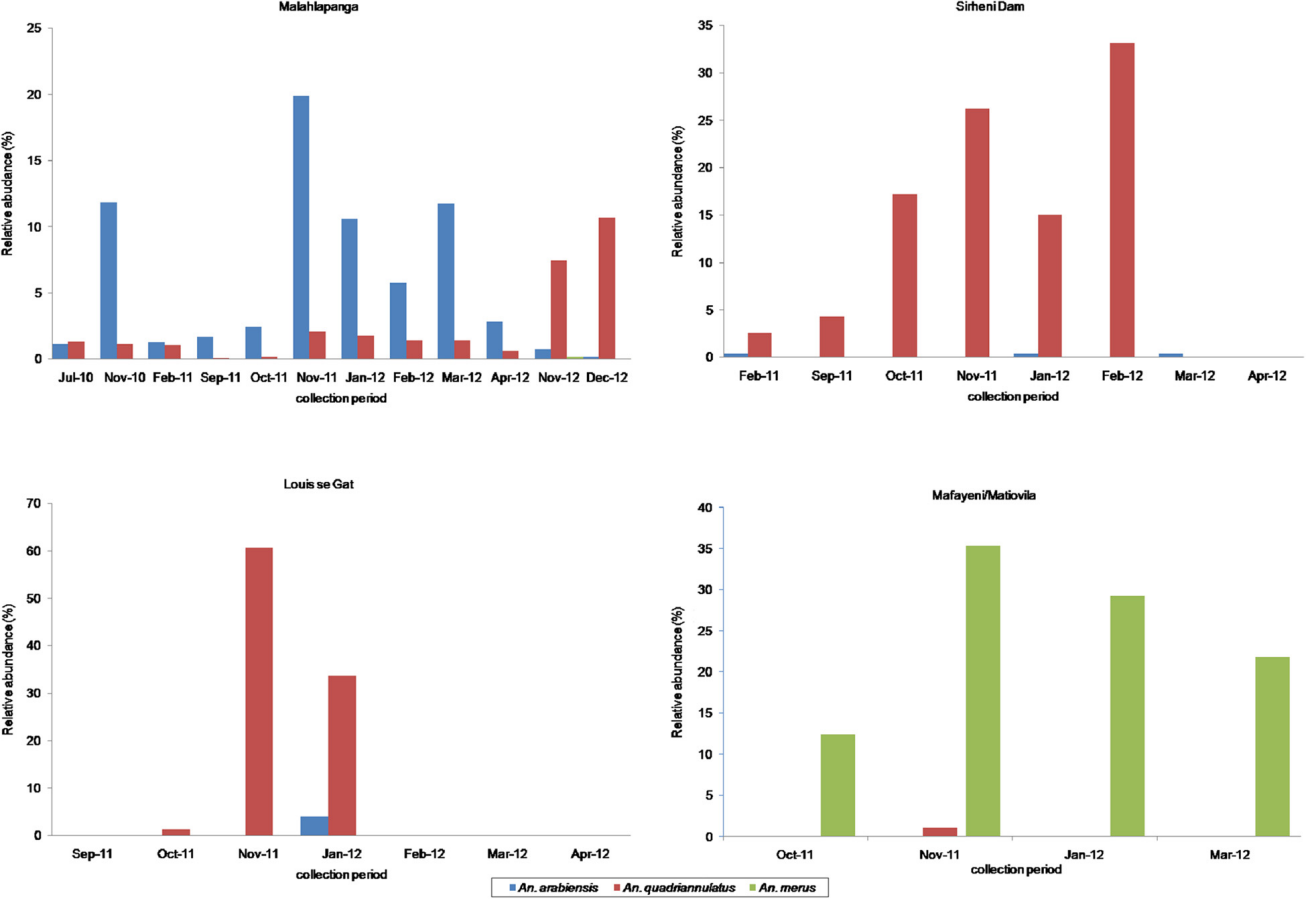
**Relative abundances of *Anopheles gambiae *** **complex specimens from five localities in northern Kruger National Park, South Africa.**

Among the nine species collected, *An. quadriannulatus* was found at four out of the five sites while *An. coustani* group was common at three sites (Malahlapanga, Sirheni bush camp and Louis se gat). *Anopheles arabiensis* was confined to Malahlapanga except in a few instances where one specimen was collected at Sirheni in February 2011, again in January 2012 and again in March 2012, and three specimens were collected from Louis se gat in March 2012 (Figure 3). The occurrence of *An. merus* was primarily limited to Matiovila and Mafayeni with the exception of two instances, November and December 2012, when specimens were unexpectedly collected in Malahlapanga. *Anopheles squamosus* and *An. rufipes* were confined to Malahlapanga. There was a significant difference in the distribution of members of the *An. gambiae* complex across the sites (chi-square = 3095; df = 6; P < 0.05). *Anopheles merus* predominantly breeds at Matiovila and Mafayeni. These two sites contributed 98.6% of the total *An. merus* catches. Of all the *An. arabiensis*, 99.6% were collected at Malahlapanga. Salinity tests of water samples from Mafayeni and Matiovila showed that the weight of sodium chloride equivalent was 12.4 g/l and 3.7 g/l, respectively.

### Seasonal abundance of *Anopheles gambiae* complex members

**Figure 4:**
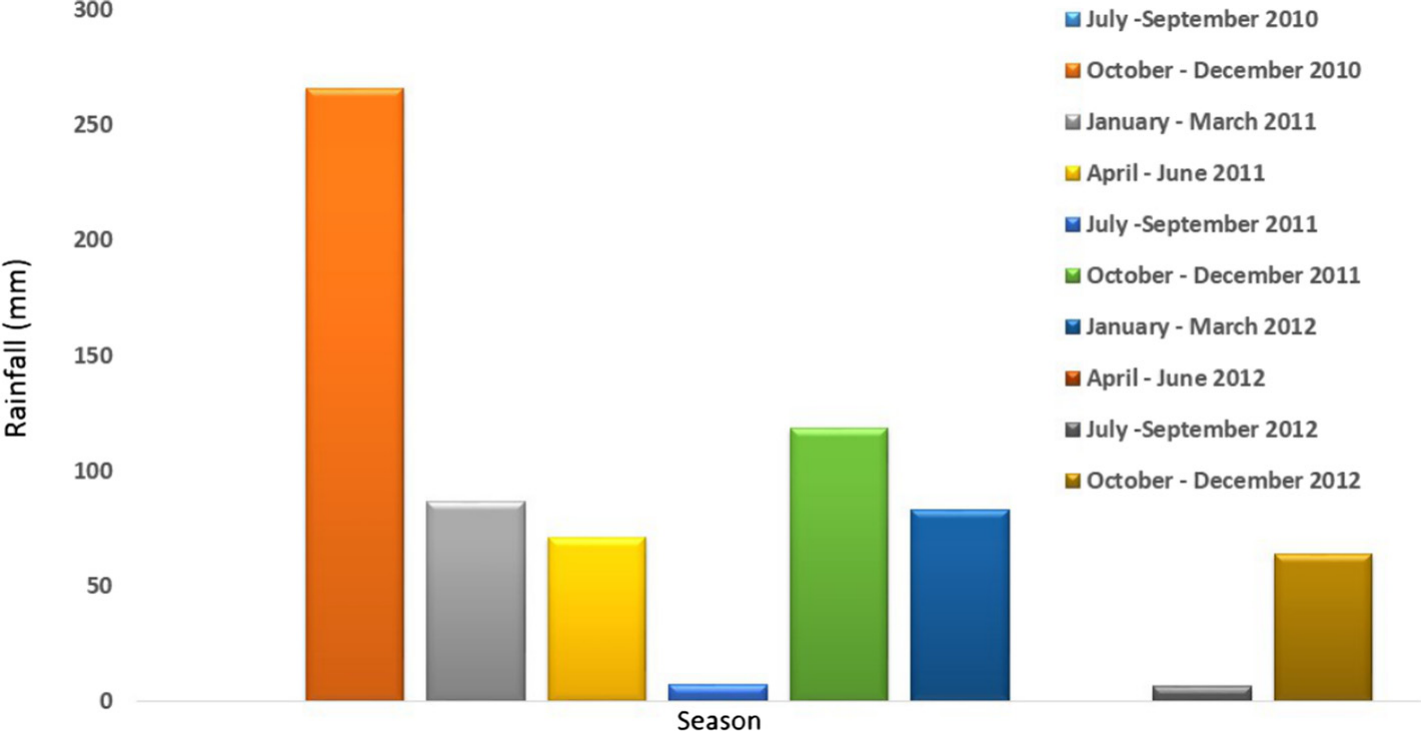
Cumulative quarterly rainfall data from July 2010 to December 2012 for Malahlapanga, northern Kruger National Park, South Africa.

The results of regular monthly surveys of members of the *An. gambiae* complex at each site are shown in Figure 3. A total of 2,571 *An. gambiae* complex specimens were collected during the sampling period. Of these 52.6% were *An. arabiensis*, 33.8% *An. quadriannulatus* and 13.6% *An. merus*. Generally mosquito abundance peaked in November regardless of species and collection site. The differences in densities were significant between seasons (repeated ANOVA, F = 6.51; P = 0.001). Abundance then steadily declined from January through to April. The lowest number of specimens was recorded during winter and the dry months of September and October 2011. This trend coincided with the rainfall pattern (Figure 4).

A total of 1,916 specimens were collected at Malahlapanga. Overall, *An. arabiensis* was the most frequently collected species followed by *An. quadriannulatus*. The *An. arabiensis* population abundance was highest in November and lowest during winter and the drier months. *Anopheles quadriannulatus* abundance was comparatively constant throughout the sampling period. Only five specimens of *An. merus* were collected over the collection period, four in November 2012 and one in December 2012. The population dynamics at Malahlapanga changed in November and December 2012, during which exceptionally few specimens of *An. arabiensis* were collected (14 and four, respectively). There was a sudden proliferation of the *An. quadriannulatus* population which had previously been present in substantially lower numbers.

At Sirheni, *An. quadriannulatus* was the most predominant species and the highest collection of this species was made in February 2012. Only three specimens of *An. arabiensis* were collected at Sirheni during the 8 months sampling period.

A total of 348 mosquito specimens was collected from Mafayeni and Matiovila. Data from these two sites were combined as they lie within 1 km of each other and have similar ecological conditions. *Anopheles merus* was the predominant species collected from these two sites contributing 98.9% of collections. The remainder were *An. quadriannulatus* specimens collected in November 2011 from Mafayeni. Relative abundance data showed that *An. merus* density was low during October 2011 when the breeding pools had almost dried out. Sampling was not done at Mafayeni and Matiovila during winter due to logistical problems in accessing the sites.

At Louis se gat a total of 74 specimens was collected during a seven month sampling period. Mosquito collections were only successful on three occasions. As with other sites November was the most successful month with only *An. quadriannulatus* being collected. Only three specimens of *An. arabiensis* were collected during the seven-month sampling period.

### Influence of sampling methods on *Anopheles gambiae* complex collections at Malahlapanga

**Figure 5:**
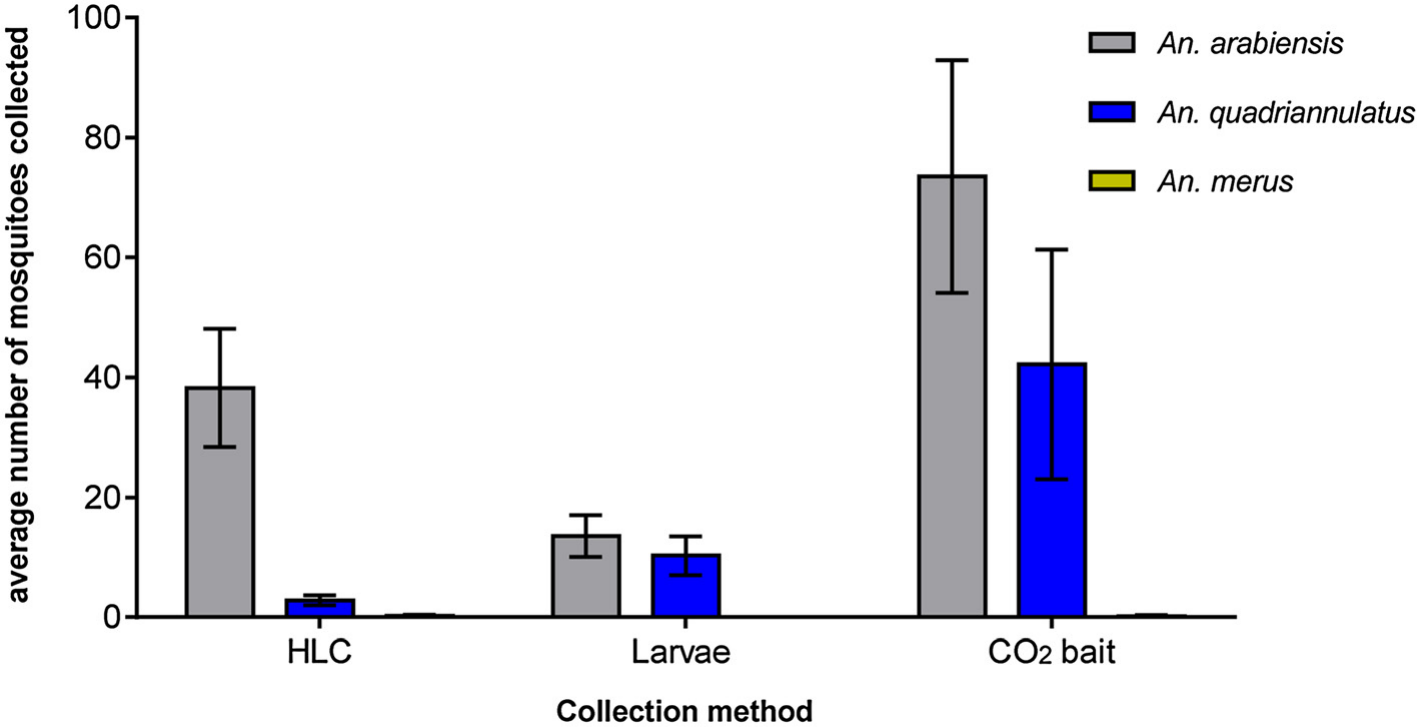
**Mean total catches per collection method of *Anopheles gambiae *** **complex specimens collected from Malahlapanga between July 2010 and December 2012.**

Analysis of the relationship between collection method and *An. gambiae* complex species composition was analysed for Malahlapanga where all three sampling methods were employed. Data for mean collections per species per collection method are shown in Figure 5. The CO_2_ trap was the most productive collection method contributing 66.5% of *An. gambiae* complex mosquitoes collected and proved good for trapping the zoophilic non-vector member species *An. quadriannulatus*. Human landing collection proved more selective for the antropophilic malaria vector *An. arabiensis* but only contributed 24.7% to the total sample size while larval collections accounted for 8.7% of the sample size. Larval collections showed the presence of both *An. arabiensis* and *An. quadriannulatus*. One way ANOVA showed that there were significant differences in mean catches per species per collection method {F (8, 81) = 6.1; P < 0.05}.

### Influence of environmental factors on mosquito catch productivity

**Figure 6:**
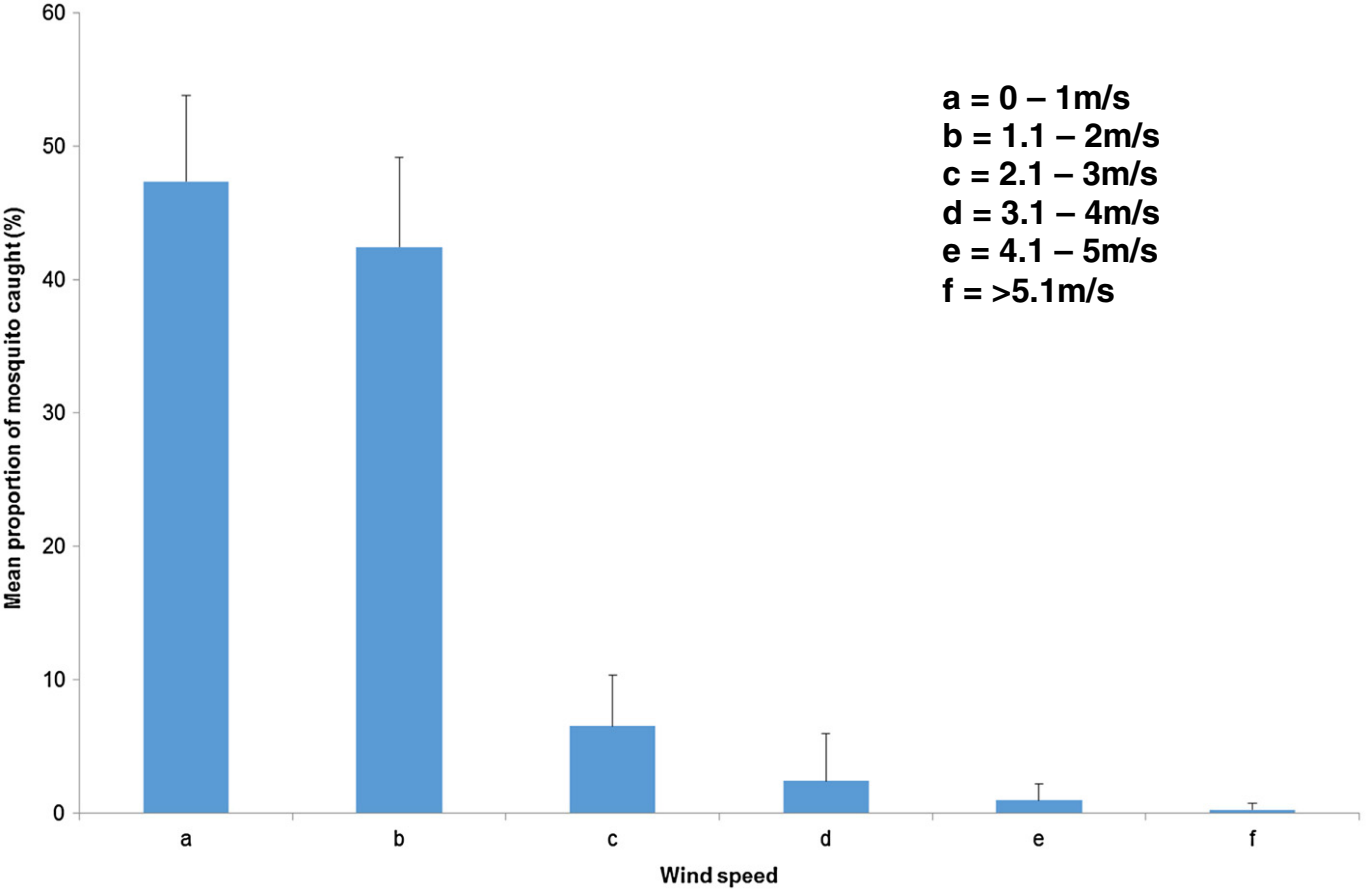
**Proportional representation of *Anopheles gambiae *** **complex caught at six different wind speeds in Malahlapanga, northern Kruger National Park, South Africa over a seven month sampling period (February, September and November 2011 and January to April 2012).**

Humidity, rainfall, temperature, and wind speed were important factors in explaining the total catches (Table 2). There was a linear relationship between mosquito productivity and these environmental variables (Multiple regression; F = 6.46; P <0.05) and 26.9% of mosquito productivity can be accounted for by humidity, temperature and wind speed (Table 2). Pearson correlation analysis showed a very strong negative correlation between wind speed and total adult catches (Figure 6). As wind speeds decreased the number of mosquitoes collected increased (Figure 4), (R^2^ = 0.8). Temperature showed a moderate positive correlation with total number of mosquitoes caught (Pearson correlation coefficient, R^2^ = 0.68). Of the three weather conditions humidity showed the lowest correlation coefficient (R^2^ = 0.5).

**Table 2:** **Summary of multiple regression analysis on *Anopheles gambiae * complex productivity in Malahlapanga, northern Kruger National Park, South Africa in relation to temperature, humidity and wind speed over a seven month sampling period (February, September and November 2011 and January to April 2012)**

	**Coefficient**	**Standard deviation**	**T**	**P**
Constant	23.7	18.7	1.27	0.312
Temperature	11.7	4.8	1.38	0.04
Humidity	7.3	16.9	2.56	0.03
Wind speed	−4.89	2.6	4.83	0.002

R^2^ = 26.9%; Adjusted R^2^ = 22.8%; F = 6.46; P < 0.05.

## Discussion

This study constitutes the first published cross-seasonal anopheline survey in the northern Kruger National Park. Nine different *Anopheles* species were collected during the sampling period. PCR identification of members of the *An. gambiae* complex showed that *An. quadriannulatus* has the widest distribution, occurring at all five sites sampled, while *An. arabiensis* was the predominant species. The wide distribution of *An. quadriannulatus* observed in this survey tallies with previous studies conducted in the Kruger National Park [11]. *Anopheles arabiensis* was mainly confined to Malahlapanga except in a few instances during the peak of the rainy season when it was found at Sirheni Dam and Louis se gat. Species-specific identification of the *An. funestus* group showed the presence of *An. rivulorum* at Malahlapanga and Sirheni Dam.

### Species distribution between collection sites

Malahlapanga showed the highest species richness with all nine species recorded there. Sirheni bush camp showed the next highest species diversity (six out nine species collected) while Matiovila and Mafayeni showed the lowest species diversity. The prevalence and distribution of anophelines in the northern Kruger National Park could be explained by the ecological conditions at each collection site. Breeding site availability, animal host availability and the presence of suitable vegetation as a source of carbohydrate (nectar) affects the presence and abundance of mosquitoes [19-21]. Of the sites sampled in this study, Malahlapanga offers the best ecological conditions for mosquito breeding. Malahlapanga contains numerous suitable breeding ponds formed by water flowing downstream from the eye of a natural hot spring. In addition, there are abundant ruminant and antelope herds that use the spring as a water source, providing a blood source for host seeking female mosquitoes. There are also perennial mosquito breeding sites at Sirheni. At Louis se gat mosquito breeding seems to occur in temporary rain puddles formed around the Mphongolo River, although the river was dry during most of the sampling period. Mosquito collections at Louis se gat were only productive during the rainy season from November through to February due to the nature of these temporary breeding pools. Limited species diversity at Matiovila and Mafayeni can be attributed to unfavorable breeding conditions. Results of water tests from these two pools showed that the water was brackish. These salinity levels are lower than those reported by others [14,22] and might explain the presence of *An. quadriannulatus* at these two sites. However, it is unclear if the salinity changes during the year as this was not measured in the current study.

### Seasonal changes in *Anopheles gambiae* complex density

Results of these surveys showed that anopheline density in the northern Kruger National Park is seasonal, with the abundance of mosquitoes peaking at the beginning of summer (rainy season). There was seasonal variation in *An. arabiensis* abundance where numbers increased dramatically following the first rains. The population number then stabilized and then significantly decreased during the dry months. During the collection period there was a second peak in abundance in late summer. This can be attributed to fluctuation in rainfall that decreased prior to a second rainy period in April. These seasonal dynamics changed during November and December 2012. In these months there was a dramatic and unexpected reduction in *An. arabiensis* abundance at Malahlapanga and an increase in *An. quadriannulatu*s abundance. In addition, three other anopheline species (*An. squamosus*, *An. rufipes* and *An. merus*) were also recorded from this site.

Reasons for this sharp change in species composition at Malahlapanga are unclear, but may have been caused by delayed rains experienced during 2012, resulting in the migration of mosquitoes in search of favourable breeding sites. It is also possible that unusually dry conditions in November-December 2012 resulted in unfavorable saline conditions at the spring. This could have been caused by a lack of groundwater recharge from rainfall at the eye of the spring and evaporative water loss at the surface. During November-December 2012, *An. merus* were recorded from Malahlapanga for the first time. The presence of *An. merus* during this time further supports the notion that a change in the salinity of the breeding sites made them unsuitable for *An. arabiensis*, but still suitable for *An. quadriannulatus* and other species recorded during this time. It will also be interesting to establish whether this sudden change in anopheline species composition and population dynamic is a permanent change or if the *An. arabiensis* population will recover over time. This highlights the importance of long-term mosquito surveillance before implementation of an intervention programme.

Another interesting phenomenon observed during this two-year mosquito survey was that collections were highly influenced by prevailing climatic conditions. Three environmental factors (humidity, temperature and wind speed) determined the number of anopheline specimens collected. Generally, humidity above 65%, temperatures above 24°C and wind speeds below 2 m/sec offered the best collection conditions. However, high humidity (85%) provided the most conducive conditions for mosquito collections. This observation is supported by other studies that show that mosquito activity is disrupted by changes in environmental conditions. Snow [23] showed that biting activity of *An. melas* and *Culex thalassius* ceases at wind speeds above 1.2 m/s and, in a similar study from South Africa, it was shown that activity of *An. merus* is greatly affected by environmental factors such as temperature, wind speed and rain [24]. Gilles and Wilkes, [25] also showed that wind has a direct effect on mosquito flight. During these collections mosquito activity decreased as a result of an increase in rain drizzle intensity. These weather conditions should be taken into consideration in order to maximize surveillance activities when vector numbers are low, especially in South Africa.

### Relationship between species composition and collection method

Analysis of the relationship between collection method and species composition was limited to Malahlapanga where all three sampling methods were productive in collecting mosquitoes. It was generally established that CO_2_-baited net traps were the most effective adult mosquito sampling technique accounting for the majority of total mosquitoes collected. However, its main disadvantage was that it was not selective for the malaria vector *An. arabiensis* and collected any host seeking female mosquito regardless of taxon. Large numbers of untargeted culicines and *Aedes* specimens were collected in the CO_2_ traps. This method is therefore most suitable for studying species diversity in an area rather than for the collection of specific taxa. Human landing collections were highly effective for collecting *An. arabiensis* females. This was an interesting observation as this population does not normally interact with humans and mainly feeds on game animals, indicating the opportunistic feeding behavior of this species. *Anopheles quadriannulatus* are generally not attracted to humans as was evident in these data. Relative species abundances based on adult collection methods do not necessarily compare with those from larval collections. Larval collections are however invaluable in terms of obtaining large samples for routine surveillance.

### Field site selection for a pilot SIT feasibility study directed against *Anopheles arabiensis*

There are a number of factors that need to be considered when choosing an appropriate site for SIT [6]. As the primary objective of this study was to choose an appropriate site to assess population reduction of *An. arabiensis*, only limited factors were considered. The first determinant was the presence of an *An. arabiensis* population in significant numbers. Ideally, the population should be geographically isolated to avoid confounding factors such as reinvasion from surrounding populations. It has been shown that an isolated mosquito population can be controlled by SIT [26,27] unless invasion from surrounding populations causes a reduction in efficacy [28,29]. The other prerequisite investigated was easy accessibility to the site. The logistics of transporting irradiated males for release and the frequency of site visits to monitor progress in population reduction are cited as important factors to consider for successful implementation of the control programmes [6].

Five areas in the northern Kruger National Park were evaluated for the presence of *An. arabiensis* populations. Of the five sites investigated, *An. arabiensis* were consistently found at Malahlapanga making it an attractive site for a SIT pilot study in South Africa. Furthermore, Malahlapanga is geographically isolated and inaccessible to tourists visiting the Kruger National Park. The nearest human habitation is approximately 9 km away making this mosquito population relatively free from human intervention. Insecticide susceptibility studies carried out on samples collected from this site showed that the population is still susceptible to all classes of insecticides [Munhenga, unpublished data]. Due to the successful malaria control programme in South Africa, there is only one other relatively large *An. arabiensis* population to be found in the country (at Mamfene in northern KwaZulu-Natal). However, extensive sampling in 2005 [30] showed a relatively small *An. arabiensis* population at Mamfene compared to the population at Malahlapanga. The greatest drawback of Malahlapanga is that it is not easily accessible throughout the year and indications from this long term surveillance showed that the *An. arabiensis* population fluctuates dramatically with no warning. Although it is only 62 km from Shingwedzi Research Camp, the greater part of the road leading to the site is smectite clay soil that makes accessibility to the site more challenging in periods of heavy rain.

## Conclusions

Anopheline species composition in the northern Kruger National Park varies by geographical location. Members of the *An. gambiae* complex occur across this region depending on habitat. Of the five sites sampled, Malahlapanga and Sirheni Dam had the highest anopheline species diversity due to the perennial availability of suitable breeding sites. Mosquito population density fluctuated with seasonal weather dynamics. Prevailing weather conditions, especially wind speed, influenced the productivity of mosquito sampling. Malahlapanga supported a perennial and geographically isolated population of *An. arabiensis* that presents a unique opportunity for assessing SIT as a malaria vector control option in a small pilot study. However, this site is not suitable for evaluating the effect of SIT on malaria transmission due to the lack of local transmission in this part of the Kruger National Park.

## Competing interests

The authors declare that they have no competing interests.

## Authors’ contributions

GM carried out fieldwork, species-specific identification and wrote the first and subsequent drafts of the manuscript. BDB was involved in fieldwork, experimental design, helped in the interpretation of results and contributed to the writing of the manuscript. BS helped with mosquito identification through morphology and PCR and contributed to the writing of the manuscript. LE was involved in fieldwork and carried out species-specific identification. RHH was involved in fieldwork, morphological identification of anophelines and provided comments on the manuscript. SM was involved in the implementation of the project and contributed to the writing of the manuscript. DG and LB were involved in the implementation of the project. LLK conceived the project, oversaw its implementation, assisted with fieldwork, species identification and contributed to the subsequent writing of the manuscript. All authors read and approved the final version of the manuscript.
